# The effect of whole-body vibration on glucose and lipid profiles in type-2 diabetes: a systematic review and pairwise and network meta-analyses of randomized trials

**DOI:** 10.1038/s41598-024-63316-0

**Published:** 2024-05-31

**Authors:** Maryam Binesh, Fatemeh Ehsani, Fatemeh Motaharinezhad, Ahmad Jayedi, Alireza Emadi

**Affiliations:** 1 Neuromuscular Rehabilitation Research Center, Semnan University of Medical Sciences, Semnan, Iran; 2 Department of Physical Therapy, School of Rehabilitation, Semnan University of Medical Sciences, Semnan, Iran; 3 Department of Occupational Therapy, School of Rehabilitation, Semnan University of Medical Sciences, Semnan, Iran; 4 Social Determinants of Health Research Center, Semnan University of Medical Sciences, Semnan, Iran; 5 Food Safety Research Center (Salt), Semnan University of Medical Sciences, Semnan, Iran

**Keywords:** Chronic disease, Glycated hemoglobin, Whole-body vibration, Diseases, Endocrinology, Health care, Medical research

## Abstract

Whole-body vibration (WBV), a training method based on the stimulation of muscle contraction by mechanical vibration generated in a vibrating platform, is claimed to be effective in diabetes management. This meta-analysis evaluated WBV effects against other exercises, placebo, or no intervention in type-2 diabetes. Medline, Scopus, and Web of Science databases were systematically searched through June 2023. Randomized controlled trials reported the effect of WBV on glucose (hemoglobin A1C and fasting blood glucose), and lipid profiles (total cholesterol, triglycerides, high, and low-density lipoprotein) were included. Two researchers independently extracted the characteristics of the studies, participants, WBV intervention and comparisons, and the outcomes from the included articles. The Physiotherapy Evidence Database (PEDro) scale assessed trial quality. In this review, all articles had no high risk of bias according to the PEDro scale, with studies achieving optimal, excellent, and good scores. Network meta-analysis revealed that WBV was effective for reducing hemoglobin A1C when compared with conventional (mean difference: − 1.58%, 95%CrI: − 2.51, − 0.47) and resistance exercise (mean difference: − 1.32%, 95%CrI: − 1.96, − 0.33). WBV had also a desirable but insignificant effect on hemoglobin A1C compared to stretching and balance exercises, placebo, and no intervention. The current pairwise meta-analysis did not show that WBV favors fasting blood glucose and lipids. WBV may have potential advantages for glycemic control in type-2 diabetes. However, uncertainties in the findings remain due to the limited number of studies and their heterogeneity.

## Introduction

Type-2 diabetes is one of the most common chronic diseases caused by the body's failure to produce or use the insulin hormone. Consequently, carbohydrate, fat, and protein metabolism are affected, and the plasma glucose level, glycosylated hemoglobin (hemoglobin A1C or HbA1C), and blood lipids increase in laboratory tests^1^. Chronic metabolic changes of diabetes are associated with multiple debilitating microvascular and macrovascular complications such as retinopathy, nephropathy, neuropathy, and cardiovascular, peripheral vascular, and cerebrovascular diseases^2^. These issues threaten different aspects of people's lives and impose a huge care burden on healthcare systems^3^.

Taking medicine, following a healthy diet, exercising, and engaging in physical activity are essential to achieving metabolic control and preventing diabetes-related problems^4^. Regular exercise reduces blood glucose, insulin resistance, and fat metabolism disturbances and protects the body against diabetes complications^5,6^. Whole-body vibration (WBV), an exercise method widely used nowadays, includes performing static and dynamic exercises on a vibrating platform^7^. It assumes that, like other exercises, WBV can effectively achieve glycemic goals and improve blood lipid control^8–10^.

Different studies investigated the effect of WBV on the metabolic parameters of diabetes; some of their data synthesis and meta-analyses are available ^11,12^. A previous meta-analysis noted that WBV can improve fasting blood glucose in people with type-2 diabetes^12^. However, this review included only a few studies and did not report other metabolic outcomes. More studies and updated meta-analyses of clinical trials are still needed to make clinical decisions about using WBV in diabetes management. The purpose of this systematic review and meta-analysis was to investigate the effect of WBV compared to other exercises, placebo, or no intervention on glucose (hemoglobin A1C and fasting blood glucose) and lipid profiles (total cholesterol, triglyceride, high-density lipoprotein, and low-density lipoprotein) in type-2 diabetes individuals.

## Methods

This study follows the Preferred Reporting Items for Systematic reviews and Meta-Analyses (PRISMA) extension statement for reporting systematic reviews incorporating network meta-analyses^13^, and its protocol was registered in International Prospective Register of Systematic Reviews (PROSPERO) (Registration number: CRD42023438514).

### Search strategy

Two researchers separately searched PubMed, Scopus, and Web of Science databases through June 7, 2023. The search was performed using the keywords of study design (randomized clinical trial), participants (people with type-2 diabetes), and intervention (whole body vibration) and their synonyms using medical subject heading keywords. All databases were systematically searched simultaneously. The literature search was developed and conducted by M.B. and A.E. Additionally, a team of two reviewers (M.B and F.M) independently screened duplicate titles, abstracts, and full-text articles. In addition, the reference list of articles included in this review and the list of references included in previous similar reviews were manually searched to identify further pertinent studies. The full search strategy used to identify original research articles for inclusion in the current systematic review is detailed in Supplementary Table 1.

### Eligibility criteria

After removing duplicate articles in the Endnote 7 software (Thomson Reuters, New York, NY, USA), two researchers separately screened the title, abstract, and, if required, the full text of the searched articles based on the following criteria. The inclusion criteria were:

- Type of participants: people with type-2 diabetes
- Type of intervention: whole-body vibration
- Type of comparison: any exercise, placebo, or no intervention
- Outcomes: hemoglobin A1C, fasting blood glucose, total cholesterol, triglycerides, high-density lipoprotein, low-density lipoprotein, and
- Study design: randomized controlled trial.

Articles in which the total WBV intervention duration was less than 6 weeks^14^ or had unclear or unreported data unavailable through the corresponding author were excluded from the review.

### Quality evaluation of included articles

Two researchers evaluated the quality and risk of bias of articles included in this review using the Physiotherapy Evidence Database (PEDro) scale. This 11-item scale measures the methodological quality of clinical trials in terms of clarity of inclusion criteria, random allocation of samples to groups, concealment of allocation, homogeneity of groups before receiving the intervention, blindness of samples, therapists, and evaluators, having less than 50% of samples dropping out, measurement of at least one key outcome and reporting their point and variability measurement, reporting of between-group statistical comparisons, and intention-to-treat analysis. Accordingly, each article is graded from 0 to 10, and a higher score indicates its higher quality^15^.

### Data extraction

Two researchers independently extracted the following information from the included articles:

- Characteristics of the participants: sample size and the mean age of the participants
- Characteristics of WBV intervention: length of intervention (week) and its frequency (number of sessions per week), intensity, magnitude, and frequency of vibration, and duration of each session
- Characteristics of the comparison group: type of intervention of the control group, length (week) and frequency (number of sessions per week) of intervention, and duration of each session
- Outcomes: times of study outcome evaluation, and mean changes and standard deviations of each outcome, including hemoglobin A1C (as the primary outcome), fasting blood glucose, total cholesterol, triglycerides, high-density lipoprotein, and low-density lipoprotein (as the secondary outcomes) in the intervention and control groups
- Characteristics of the study: first author and year of publication.

The mean and standard deviation were calculated using standard Cochrane formulas in the articles that reported the data as standard error, interquartile range, or confidence interval^16^. In articles with unclear or missing information, the corresponding author was requested to provide the data by email. Any disagreements between the researchers in the study's screening, data extracting, or article quality evaluation stages were resolved by agreement through discussion with a third researcher.

### Data synthesis and analysis

Data analysis was conducted using Stata software version 17. Pairwise meta-analysis was conducted using a random-effects model to compare WBV with no intervention or placebo on fasting blood glucose, total cholesterol, triglyceride, and high and low-density lipoprotein. The findings were reported as the mean difference with a 95% confidence interval. Network meta-analysis with a random-effects model was used for multiple comparisons between the WBV and any exercise, no intervention, or placebo on hemoglobin A1C. The ranking probability of each intervention from all included studies was reported as the surface under the cumulative curve (SUCRA) values from zero to one. Accordingly, the intervention with a more significant SUCRA value was more effective^17^. Heterogeneity in each pairwise comparison was estimated using the I^2^ statistic; values greater than 25% were considered significant heterogeneity between the studies' results^18^. The generalized inconsistency test also assessed network heterogeneity between studies; a significance level greater than 0.05 indicates consistency^19^. Due to the small number of studies included in the analysis, certainty of the evidence for each direct comparison according to standard Grades of Recommendation, Assessment, Development, and Evaluation (GRADE) guidance was not assessed^20^. We also could not conduct a network meta-analysis for secondary outcomes.

### Ethical approval.

Not applicable.

## Results

### Systematic search and study selection

Supplementary Fig. 1 illustrates the process of literature searches and study selection. The initial systematic search led to the identification of 289 studies (Supplementary Table 1). There were 243 duplicate articles, and 216 did not qualify according to their titles and abstracts. After adding three trials in the manual search of the reference list of included articles, the study was completed with the analysis of ten papers^21–30^ that met our inclusion criteria.

### Characteristics of the included trials

A total of 428 participants was examined in ten trials included in the present meta-analysis. These trials were made available from 2007 to 2021. The percentage of male and female subjects was not reported in some studies^25,30^. In one study^27^, the mean age for all groups was over 70 years, whereas participants in other studies tended to be younger than 70 years old. The hemoglobin A1C level of participants in all trials was less than 10. In 9 trials, the intervention group received only WBV^21–26,28–30^, while one trial delivered WBV combined with balance exercise^27^. Two trials delivered WBV in six weeks^26,27^, two trials in 8 weeks^23,25^, and the others in 12 weeks. Table 1 shows the characteristics of the trials included in this meta-analysis.

**Table 1:** Characteristics of randomized clinical trials included in the systematic review and meta-analysis of the effect of whole-body vibration on glucose and lipid profiles in type-2 diabetes

Studies	Participants N (Sample size)	Intervention group		Control group		Time of assessments	Outcomes
		Intervention	Duration	Intervention	Duration		
Michels et al., 2021, Brazil^29^	N = 24 (IG = 12, CG = 12)/ 59.18 (± 6.40) years/ 68.2% female	Diet and physical activity + daily WBV a fixed frequency of 28 Hz, maximum intensity of 0.6 g in the biped position for a period of 20 to 30 min daily	12 weeks, Daily, 20–30 min	Advice on adopt routine diet and physical activity	–-	Before and at the end of 12-week intervention	Hemoglobin A1C, Fasting Blood Glucose, Total cholesterol, Triglycerides, HDL cholesterol, LDL cholesterol
Ramachandran et al., 2021, India^30^	N = 36 (Group A = 12, Group B = 12, Group C = 12)/ 51.2 (± 2.3) years/ % female not reported	WBV amplitude 2 mm and 30 Hz of vibration frequency for nine weeks. From 10 to 12th week the frequency increased 35 Hz for the duration of 20 min	12 weeks, 3 days per week, 20 min	Group B: resisted exercise Group C: usual walking	12 weeks Group B: 3 days per week Group C: 5 days per week	Before and at the end of 12 week intervention	Hemoglobin A1C, Triglycerides, HDL cholesterol, LDL cholesterol
Domínguez-Muñoz et al., 2020, Spain^25^	N = 90 (IG = 45, CG = 45)/ mean age and % female not reported	WBV with a knee flexion at 45 during five to nine series of 30–60 s in a vibration frequency that oscillated between 12.5 and18.5 and 30 s of recovery between series	8 weeks, 3 times per week, 12–27 min per week	Placebo: The same protocol but without vibration	8 weeks, 3 times per week, 12–27 min per week	Before and at the end of 8 week intervention	Hemoglobin A1C, Fasting Blood Glucose r, Total cholesterol, HDL cholesterol, LDL cholesterol
Ahmed et al., 2019, Saudi Arabiay^21^	N = 60 (IG = 30, CG = 30), 43.96 (± 3.09) years/ 100% female	WBV + Exercise program in two sets of six positions	12 weeks, 3 sessions per week, 36 min per week	Moderate-intensity aerobic cycling exercises on a stationary bicycle	12 weeks, 3 times per week, 30–40 min each session	Before and at the end of 12 week intervention	Hemoglobin A1C
Manimmanakorn et al., 2017, Thailand^28^	N = 40 (IG = 20, CG = 20), 63.2 (± 7.7) years/ 65% female	Diet, physical activity, and prescribed medications + WBV for two sets of 6 one-minute vibration squats interspersed with 20 s rest periods, 3 times per week for 12 weeks	12 weeks, 3 times per week, 36 min per week	Routine diet, Medication, and physical activity	–	Before and at the end of 12 week intervention	Hemoglobin A1C, Fasting blood Glucose
del Pozo-Cruz et al., 2014, New Zealand^24^	N = 50 (IG = 25, CG = 25)/ 71.6 (± 8.54) in IG, 66.8 (± 10.83) in CG / 45% in IG, 50% in CG	WBV with static and dynamic exercises, three sessions per week with at least one day between sessions. Each exercise session was performed with a frequency of 12 Hz for the first month, 14 Hz for the second month, and 16 Hz for the last month	12 weeks, 3 times per week, 8–12 min per session	Standard care	–	At baseline and after the 12-week intervention	Hemoglobin A1C, Fasting Blood Glucose, Total cholesterol, Triglycerides, HDL cholesterol, LDL cholesterol
Lee et al., 2013, Korea^27^	N = 60 (WBV + BE = 20, BE = 20, CG = 20)/ 76.31(± 4.78) in WBV + BE, 74.05(± 5.42) in BE, 75.77(± 5.69) in CG	The WBV and BE. BE comprised 10 min of warm-up activities, 40 min of balance training, and 10 min of cool-down activities. The WBV with Lower frequencies and amplitudes (15 Hz and peak-to-peak amplitude of 2 mm) were used initially. WBV was performed at a frequency of 15–30 Hz and amplitude of 1–3 mm	BE:60 min per day, 2 times per week, for 6 weeks, WBV: 3 × 3 min, 3 times per week, for 6 weeks (27 min/week)	No intervention	BE: 60 min per day, 2 times per week, for 6 weeks	at baseline and after 6 weeks of training	Hemoglobin A1C
Kordi Yousefi Nejad et al., 2013, Iran^26^	N = 20 (IG = 10, CG = 10)/ 57.3 (± 5.6) in IG,57 (± 4.8) in CG, 60% in both groups	WBV with a frequency of 30 Hz and amplitude of 2.5 mm for 5 sets of 30 s in the first two weeks of the intervention. In the third and fourth weeks, the duration of WBV was 5 sets of 45 s, and in the fifth and fourth weeks, they received 5 sets of one-minute vibrations. The rest period between sets was one minute throughout the study	6 weeks, two times per week, 2.5–5 min per session	No intervention	–-	Before and at the end of 6- week intervention	Hemoglobin A1C, Fasting Blood Glucose, Total cholesterol, Triglycerides, HDL cholesterol, LDL cholesterol
						Before (pre-test), at the end of the fourth week (midtest),	Hemoglobin A1C, Fasting Blood Glucose
	Behboudi et al., 2011, Iran^23^	N = 30 (WBV = 10, Aerobic exercise = 10, CG = 10)/ 49.2 (± 3.94) in WBV,53.1 (± 6.57) in Aerobic, 52.3 (± 6.17) in CG	WBV with 30 Hz of frequency and 2 mm of amplitude for 16 min [1 min vibration with 8 iterations and 1 min break between each iteration], 20, and 24 min in the first week to the third week, the fourth week to the sixth week, and the seventh week to the eighth week respectively	8 weeks WBV, 3 sessions per week	Aerobic exercise: an 8-week increasing aerobic exercise program with 3 sessions per week for 30–60 min. CG: No intervention	Aerobic: 8 weeks, 3 times per week	and at the end of the eighth week (post-test)
Baum et al., 2007, Germany^22^	N = 40 (WBV = 14, Strength training = 13, Stretching = 13)/ 62.2 (± 4) in WBV,62.9 (± 7.3) in Strengh T, 63.3 (± 5.9) in Stretching/ 40%	A horizontal swinging platform with an amplitude of 2 mm and a frequency of 30–35 Hz.Training sessions consisted of 8 different exercises including muscles of the whole body. Subjects were encouraged to work isometricaly against the swinging platform up to 20 min	12 weeks, 3 days per week, Up to 20 min/session	Strenth training: 8 strenghtening exercises for the upper and lower body. Stretching: 8 static exercises	12 weeks, 3 days per week	At baseline and after 12 weeks of intervention	Hemoglobin A1C, Fasting Blood Glucose

IG: Intervention group; CG: control group; BE: Balance exercise; HDL: High-density lipoprotein; LDL: Low-density lipoprotein; WBV: Whole body vibration

### Quality of the included trials

Among all the articles included in this review, no study had a high risk of bias based on the PEDro scale (Supplementary Table 2). Accordingly, five studies obtained optimal scores with a low risk of bias^21,24,25,27,28^, two of which were excellent^25,28^, while the others were good^22,23,26,29,30^.

### Network *meta*-analysis: effect of different interventions on hemoglobin A1C

**Figure 1:**
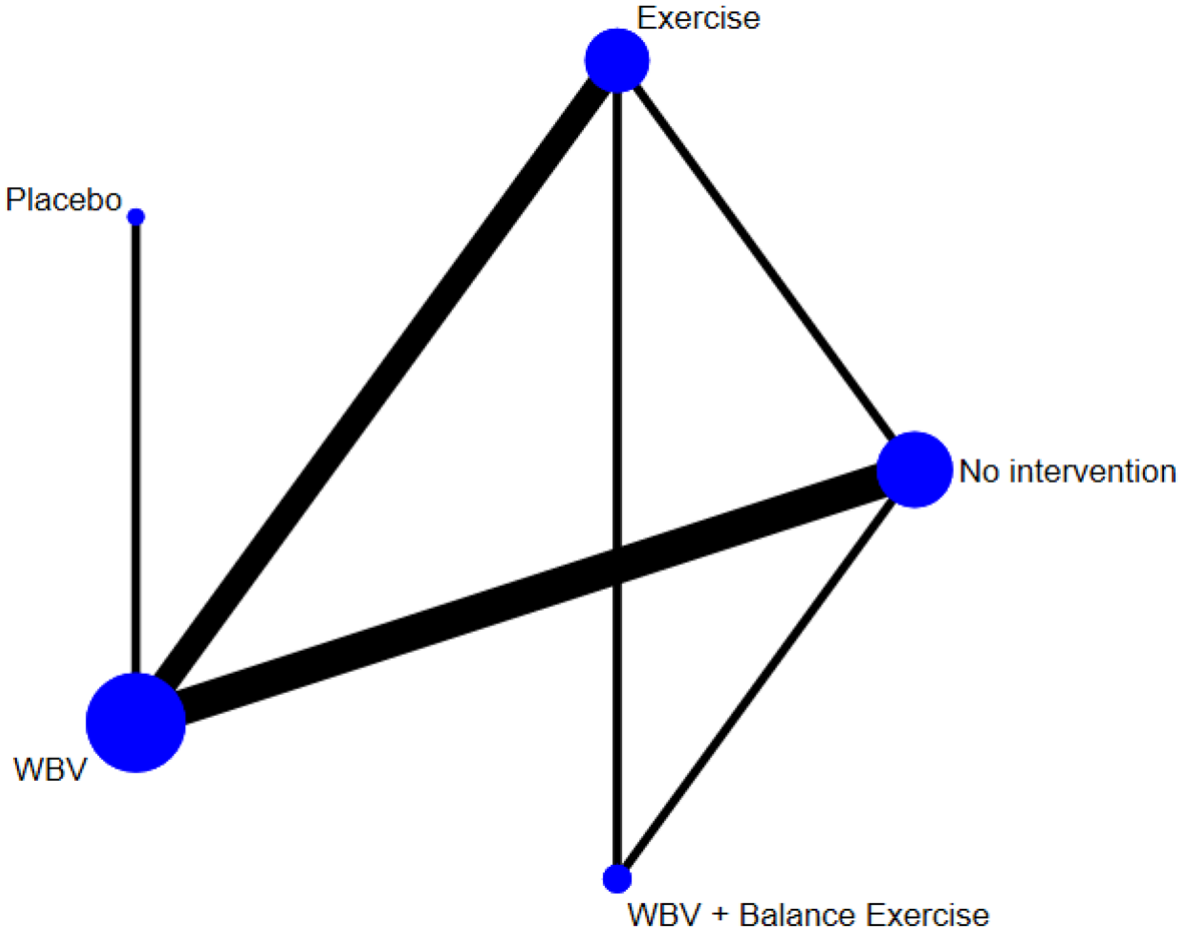
The effect of whole-body vibration on hemoglobin A1C

Figure 1 and Table 2 present the comparative effects of different interventions on hemoglobin A1C. The results indicated that WBV was effective for reducing hemoglobin A1C when compared with conventional (mean difference: − 1.58%, 95%CrI: − 2.51, − 0.47) and resistance exercise (mean difference: − 1.32%, 95%CrI: − 1.96, − 0.33). WBV also had a favorable but insignificant effect on hemoglobin A1C compared to stretching and balance exercises, placebo, and no intervention. Aerobic exercise was effective compared to conventional exercise (mean difference: − 1.64%, 95CrI: − 3.03, − 0.03). None of the other interventions were effective in reducing hemoglobin A1C when compared with placebo and no intervention nor with each other. SUCRA values for the effects of different interventions on hemoglobin A1C highlight WBV as the most effective training (Table 3).

**Table 2:** Comparative effect of different interventions on HbA1c in patients with type 2 diabetes (mean difference and 95% credible interval)

Treatments	Aerobic exercise							
Balance exercise	− 0.52 (− 2.14, 1.12)	Balance exercise						
No intervention	− 0.51 (− 1.72, 0.72)	0.01 (− 1.07, 1.08)	No intervention					
Conventional exercise	− 1.64 (− 3.03, − 0.03)	− 1.12 (− 2.62, 0.56)	− 1.13 (− 2.2, 0.13)	Conventional exercise				
Placebo	− 0.1 (− 1.64, 1.41)	0.42 (− 1.23, 2.02)	0.41 (− 0.82, 1.6)	1.54 (− 0.06, 2.92)	Placebo			
Resisted exercise	− 1.39 (− 2.56, 0.16)	− 0.88 (− 2.15, 0.78)	− 0.87 (− 1.68, 0.29)	0.23 (− 0.67, 1.37)	− 1.29 (− 2.44, 0.27)	Resisted exercise		
Stretching exercise	− 1.25 (− 2.67, 0.52)	− 0.74 (− 2.25, 1.12)	− 0.74 (− 1.88, 0.71)	0.37 (− 0.96, 1.9)	− 1.15 (− 2.56, 0.63)	0.11 (− 1.01, 1.26)	Stretching exercise	
WBV	− 0.05 (− 1.14, 1.03)	0.47 (− 0.76, 1.67)	0.46 (− 0.1, 1)	1.58 (0.47, 2.51)	0.05 (− 1.03, 1.14)	1.32 (0.33, 1.96)	1.19 (− 0.14, 2.23)	WBV
WBV and balance exercise	− 0.45 (− 2.06, 1.19)	0.07 (− 1.01, 1.14)	0.06 (− 1.01, 1.14)	1.19 (− 0.49, 2.69)	− 0.35 (− 1.95, 1.3)	0.95 (− 0.71, 2.21)	0.81 (− 1.04, 2.32)	− 0.4 (− 1.6, 0.83)

WBV: Whole-body vibration

**Table 3:** SUCRA values for the effects of types of interventions on HbA1C

Outcomes	HbA1C
Whole-body vibration	0.824
Aerobic exercise	0.822
Placebo	0.757
Whole-body vibration and Balance exercise	0.565
Balance exercise	0.509
No intervention	0.506
Stretching exercise	0.241
Resisted exercise	0.191
Conventional exercise	0.081

### Pairwise *meta*-analysis

Table 4 presents the effects of WBV on secondary outcomes in type-2 diabetes. Accordingly, WBV was ineffective in reducing fasting blood glucose, serum triglyceride, total cholesterol, and high and low-density lipoprotein (Table 4 and Supplementary Figs. 2, 3, 4, 5, 6).

**Table 4:** The effects of whole-body vibration against placebo or no intervention on secondary outcomes

Outcome	Number of trials	Number of participants	Mean difference (95%CI)	I2, Pheterogeneity
Fasting blood sugar	6	135	− 10.45 (− 22.65,1.75)	88.20% (0.09)
Total cholesterol	4	171	2.34 (-23.26,27.94)	99.84% (0.86)
Triglyceride	3	81	− 33.75 (− 69.12,1.62)	80.34% (0.06)
High-density lipoprotein	4	171	5.51 (− 4.45, 15.46)	97.78% (0.28)
Low-density lipoprotein	4	171	5.29 (− 6.64,17.22)	97.94% (0.38)

## Discussion

Type-2 diabetes is one of the most common metabolic diseases and a healthcare concern worldwide. WBV exercise has gained attention recently for its potential benefits in metabolic control and diabetes management. The evidence suggests that WBV can improve insulin sensitivity, glucose metabolism, and cardiovascular health in individuals with diabetes^11^. The purpose of this study was to write a systematic review and meta-analysis of interventions that investigated the effect of WBV on metabolic indices (glucose and lipid profiles) of people with type-2 diabetes. Interestingly, our network meta-analysis showed that compared to other exercise methods (conventional, resistance, stretching, balance, and aerobic exercises), placebo, and not receiving intervention, WBV was the most effective on glycemic control by reducing glycosylated hemoglobin.

WBV is a simple, not time-consuming, and inexpensive method that can be applied consistently by people with diabetes^11^. By affecting glycosylated hemoglobin as the most critical indicator of glycemic control in diabetes^31^, it seems that WBV helps to reduce diabetes complications and care burdens^32^. Some research focuses on WBV's potential effects on muscle activation and suggests that WBV may activate AMP-activated protein kinase (AMPK) in muscle, which is a key regulator of cellular energy metabolism. Activation of AMPK can lead to various metabolic responses in muscle cells^33^. In other words, as a strong stimulus, WBV causes the continuous and rapid lengthening and shortening of the muscle–tendon complex. Consequently, the muscle spindles are discharged and stimulate the activity of the alpha motor neuron through the monosynaptic and polysynaptic pathways, which leads to the muscles' reflex contraction under the tonic vibration reflex. Muscle contraction causes more glucose uptake from the bloodstream to the muscle cells through various mechanisms, such as reducing the resistance of liver and muscle cells to insulin, increasing glucose transportation protein (GLUT-4), increasing the density of muscle capillaries and muscle blood flow, and increasing glucose absorption by muscles^8,9,34^. Repetition of muscle contraction with vibration stimulation may also affect glycemic targets by arousing endocrine responses and regulating the balance between insulin and glucagon hormones^9,34,35^.

The current study noted a significant decrease of 1.58 and 1.32% in glycosylated hemoglobin in WBV intervention compared to conventional and resisted exercise. This promising finding is comparable to improving glycemic control through medication^36^. An increase in the gravitational load imposed on the musculoskeletal system by the vibration stimulus and, consequently, an increase in the muscle power production capacity can justify this finding^8^. The WBV also had a desirable effect on hemoglobin A1C compared to stretching and balance, exercises, but without significant differences between the intervention and controls. Of course, this lack of significance is likely due to the small number of studies available for analysis and their diversity in terms of the studied population, vibrating platform parameters, and training protocols. On the other hand, different exercise methods have a proven effect on the glycemic control of people with diabetes. Therefore, it is close to the expectation that, like the WBV, they favor reducing glycosylated hemoglobin^5^.

The strange finding of the present meta-analysis was that WBV intervention's reduction effect on glycosylated hemoglobin was insignificant compared to no intervention or placebo. However, we found only one randomized clinical trial that evaluated the effect of WBV against placebo^37^. The placebo effect of the vibrating platform, along with the attendance of the placebo group participants in the care center to receive the intervention and having the positive social support of peers and experts^38^ may explain this finding. Previous research emphasized on the development of standardized interventions to consider different parameters related to the vibration platform, such as its amplitude and frequency and the intensity of the exercises^14^. Regarding the heterogeneity of existing interventions, they seem to underestimate the importance of developing and using standardized vibration and training protocols, may have affected the results.

Unlike the previous study^12^, our study's fasting blood glucose and lipid profile analysis did not show significant differences between WBV and other controls. Notably, few studies investigated these parameters in WBV interventions. In agreement with our findings, another meta-analysis reported no effect of exercise on fasting blood glucose in type-2 diabetes^39^. Available evidence has also reported the dose–response relationship between physical exercise and blood lipid changes^40^. Accordingly, small-volume exercises favor changing blood lipids, but significantly changing these parameters depends on the intensity and dose of exercise training. Maybe the exposure time and intervention volume in the studies included in this meta-analysis were insufficient to change these indices significantly.

### The strengths of the study

Globally, the current meta-analysis highlights the main clinical benefits of WBV in type-2 diabetes. This research is the first to use network meta-analysis plus pairwise meta-analysis to compare a variety of controls with WBV. In addition to sound quality, the studies included in our analysis had the highest level of evidence, having a randomized clinical trial design.

### The limitations and suggestions

The limited number of studies available for analyzing the variables under investigation introduces some uncertainty to our findings. The existing literature has a great variety in vibration parameters, types of exercise, and training duration. Meanwhile, subgroup analysis was not possible to determine which variable contributed the most to HbA1C improvement. Upcoming research should consider poor consistency between training protocols and try to develop and implement standardized interventions with a larger sample size. Recently published guidelines can be used to introduce all the variables that must necessarily be considered in the development, implementation, and scientific reporting of WBV interventions^7^. The suggestion for future research with a larger sample size and standardized protocols is crucial for advancing our understanding and potentially unlocking new treatment options for diabetes management. In order to provide the best effects, upcoming protocols must contemplate various parameters, including optimal dose–response relationships and various vibration characteristics. Moreover, they need to use suitable methods to monitor the possible side effects of WBV.

## Conclusion

WBV can beneficially improve the hemoglobin A1C in type-2 diabetes. This exercise method seems to help reduce diabetes complications and its care burden by improving glycemic control. More well-designed future studies are needed to draw definitive conclusions about the effects of whole-body vibration on blood lipids and fasting sugar in those with diabetes.

### Supplementary Information

Supplementary Information 1.

Supplementary Information 2.

Supplementary Information 3.

Supplementary Information 4.

Supplementary Information 5.

Supplementary Information 6.

Supplementary Information 7.

Supplementary Information 8.

## Data Availability

The data used and analyzed during the current study are available from the corresponding author on reasonable request.
