# Supplementary material for: The effect of whole-body vibration on glucose and lipid profiles in type-2 diabetes: a systematic review and pairwise and network meta-analyses of randomized trials
Source: Sci Rep. 2024 May 31;14:12494. doi: 10.1038/s41598-024-63316-0 (PMC11143234; doi:10.1038/s41598-024-63316-0)
Supplement: Supplementary file 1 — Supplementary Information 1. [file 41598_2024_63316_MOESM1_ESM.docx]

## Identification

## Screening

Database searching (n=289)

Manual search (n=3)

Items after duplicates removed
(n = 243)

Items excluded
(n =216)

Title and abstract screened
(n = 243)

Articles assessed for eligibility
(n =27)

Excluded articles (n =17)

- Methodology not matched (Not- Randomized Trial, Study protocol and Compare data RCT with NRCT) (n=5)

- Population not matched (n= 3)

- Outcome not matched (n= 5)

- Intervention not matched (n= 4)

Studies included in qualitative and quantitative synthesis
(n = 10)

## Included

## Eligibility

**Supplementary Figure 1**. Literature search and study selection process
