# Supplementary figures and images for: The effect of whole-body vibration on glucose and lipid profiles in type-2 diabetes: a systematic review and pairwise and network meta-analyses of randomized trials
Source: Sci Rep. 2024 May 31;14:12494. doi: 10.1038/s41598-024-63316-0 (PMC11143234; doi:10.1038/s41598-024-63316-0)

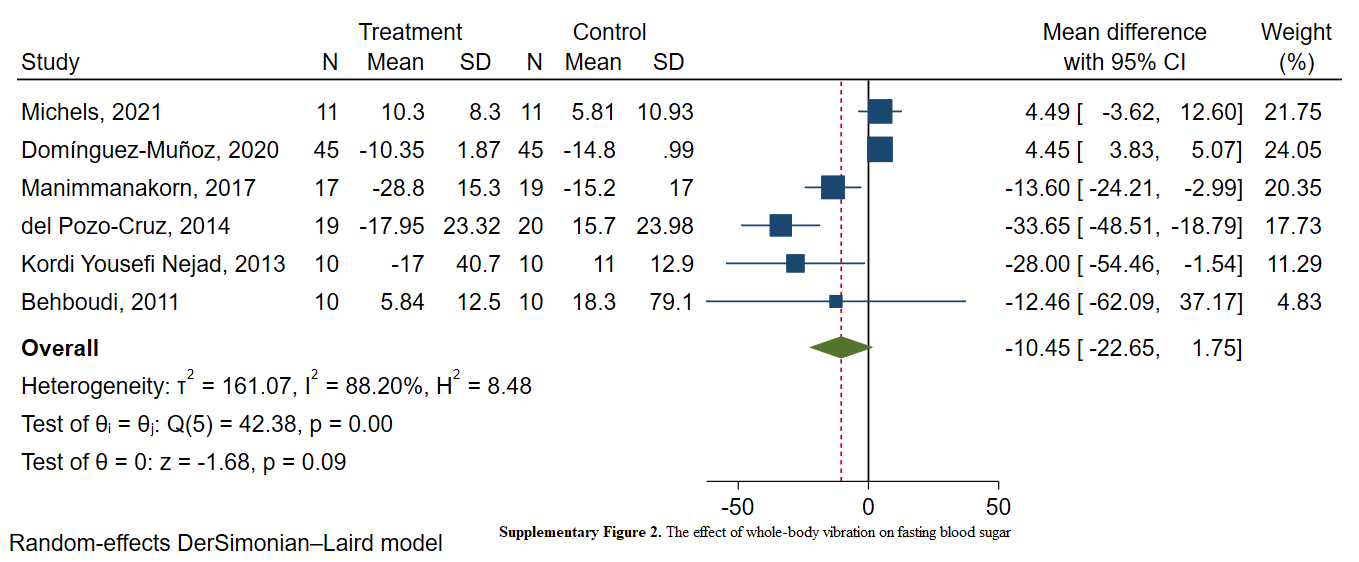

Supplement: Supplementary file 2 — Supplementary Information 2. [file 41598_2024_63316_MOESM2_ESM.tif]

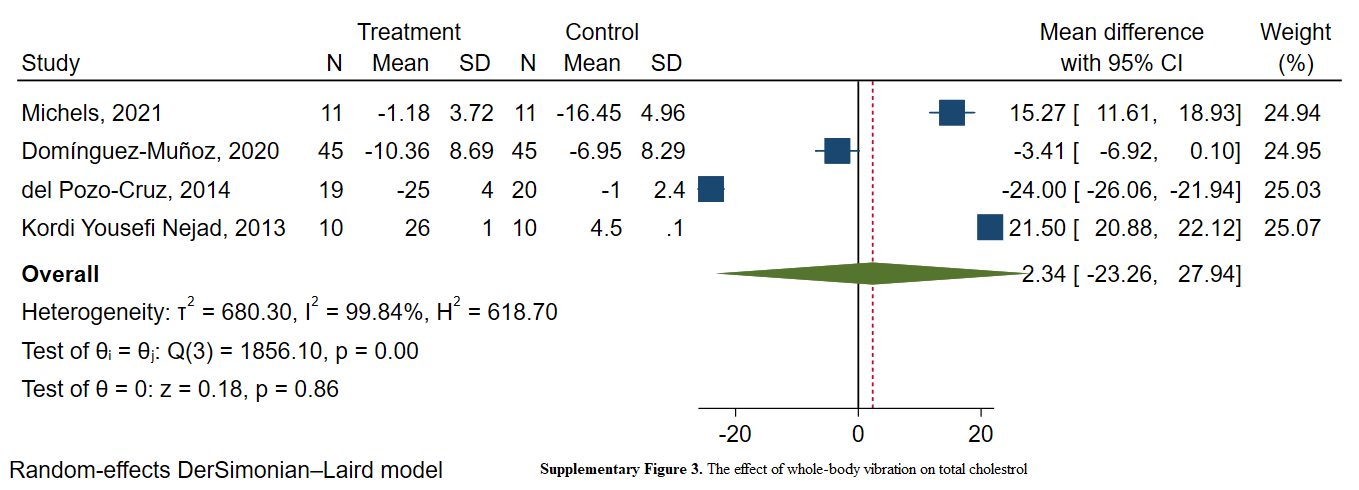

Supplement: Supplementary file 3 — Supplementary Information 3. [file 41598_2024_63316_MOESM3_ESM.tif]

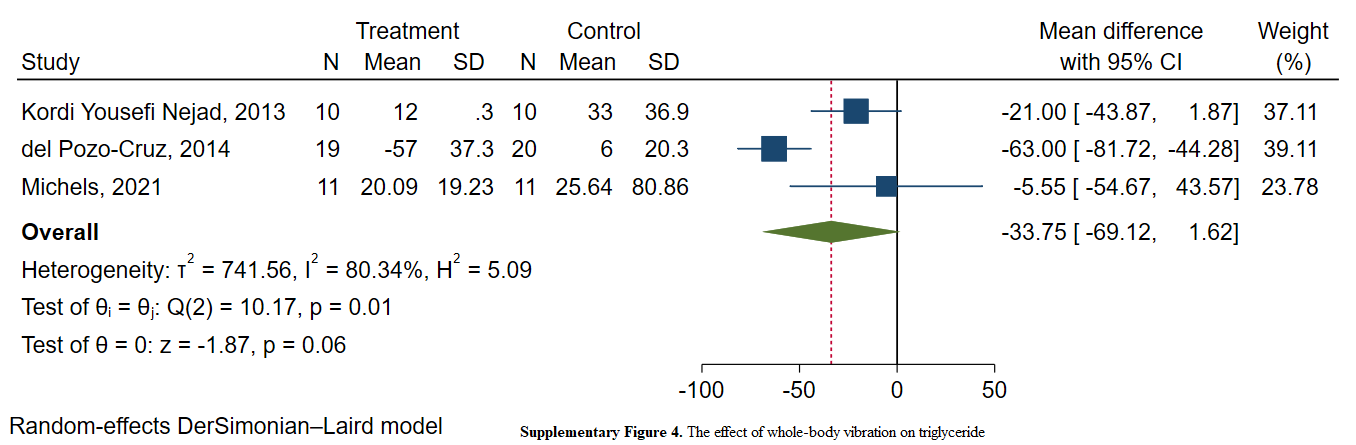

Supplement: Supplementary file 4 — Supplementary Information 4. [file 41598_2024_63316_MOESM4_ESM.tif]

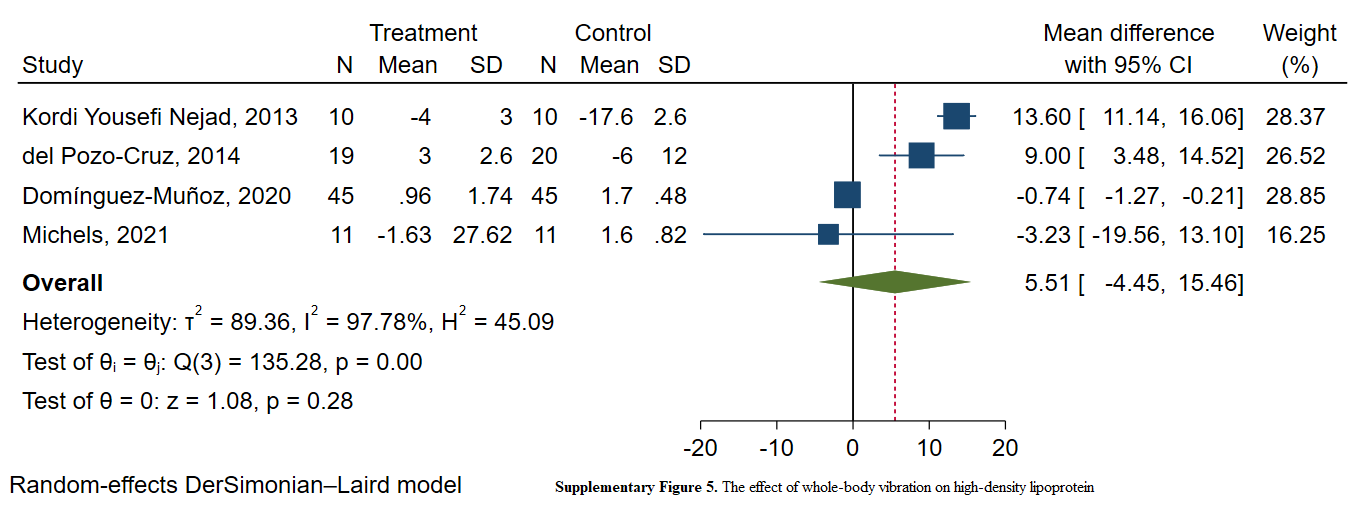

Supplement: Supplementary file 5 — Supplementary Information 5. [file 41598_2024_63316_MOESM5_ESM.tif]

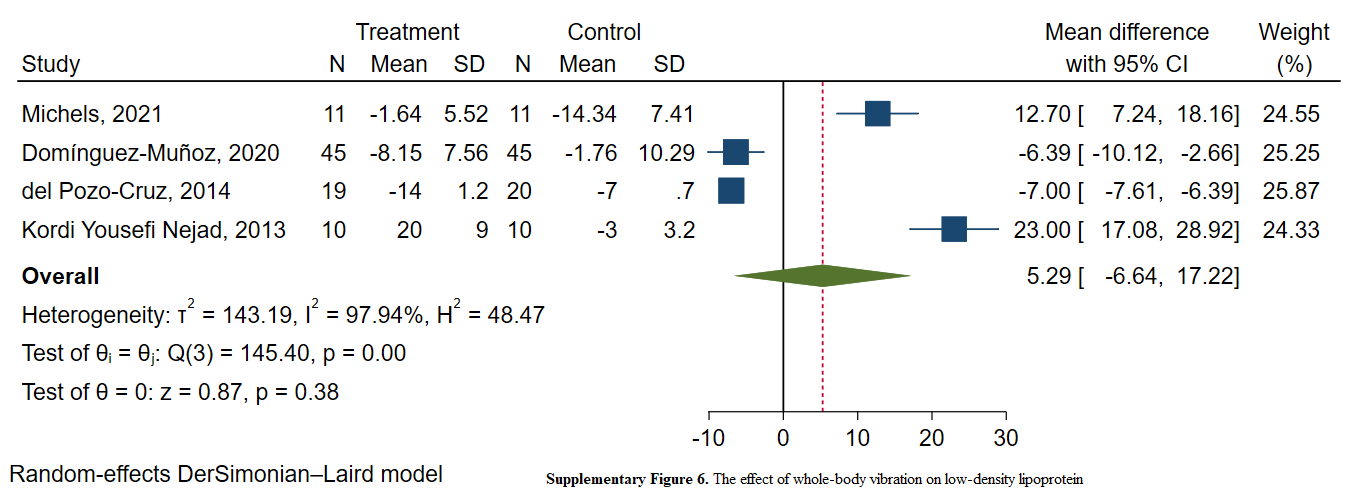

Supplement: Supplementary file 6 — Supplementary Information 6. [file 41598_2024_63316_MOESM6_ESM.tif]
