# Supplementary material for: The effect of whole-body vibration on glucose and lipid profiles in type-2 diabetes: a systematic review and pairwise and network meta-analyses of randomized trials
Source: Sci Rep. 2024 May 31;14:12494. doi: 10.1038/s41598-024-63316-0 (PMC11143234; doi:10.1038/s41598-024-63316-0)
Supplement: Supplementary file 7 — Supplementary Information 7. [file 41598_2024_63316_MOESM7_ESM.docx]

**Supplementary Table 1.** Search strategy to find potential relevant articles for inclusion in the meta-analysis of whole-body vibration in type-2 diabetes (June 7, 2023).

| **Search strategy:**  (Whole body vibration [ti/ab] OR whole body vibration [all fields]) AND (diabetes [ti/ab] OR diabetic [ti/ab] OR Diabetes Mellitus [Mesh] OR Diabetes Mellitus, Type 2 [Mesh]) OR (random* [ti/ab] OR trial* [ti/ab]) |
| --- |
| **PubMed (45)** |
| **Scopus (166)** |
| **Web of Science (n=78)** |
| **All: 289** |
