# Supplementary material for: The effect of whole-body vibration on glucose and lipid profiles in type-2 diabetes: a systematic review and pairwise and network meta-analyses of randomized trials
Source: Sci Rep. 2024 May 31;14:12494. doi: 10.1038/s41598-024-63316-0 (PMC11143234; doi:10.1038/s41598-024-63316-0)
Supplement: Supplementary file 8 — Supplementary Information 8. [file 41598_2024_63316_MOESM8_ESM.docx]

| **Supplementary Table 2.** Quality assessment of included articles | | | | | | | | | | | | |
| --- | --- | --- | --- | --- | --- | --- | --- | --- | --- | --- | --- | --- |
| PEDro Scale items | 1 | 2 | 3 | 4 | 5 | 6 | 7 | 8 | 9 | 10 | 11 | Score |
| Manimmankorn et al., 2017 (25) | + | + | + | + | _ | _ | + | + | + | + | + | 9 |
| Daminguez-Monuz et al., 2020 (22) | + | + | + | + | + | _ | _ | + | + | + | + | 9 |
| Ahmed et al., 2019 (18) | + | + | _ | + | _ | _ | + | + | + | + | + | 8 |
| Del Pozo-Cruz et al., 2013 (21) | + | + | + | + | _ | _ | + | _ | + | + | + | 8 |
| Lee et al., 2013 (24) | + | + | + | + | _ | _ | _ | + | + | + | + | 8 |
| Michels et al., 2020 (24) | + | + | _ | + | _ | _ | + | + | + | _ | + | 7 |
| Ramachandran et al., 2021 (27) | + | + | _ | + | _ | _ | _ | + | + | + | + | 7 |
| Behboudi et al., 2011 (20) | + | + | _ | + | _ | _ | _ | + | + | + | + | 7 |
| Kordi Yousephi Nejad et al., 2013 (23) | + | _ | _ | + | _ | _ | _ | + | + | + | + | 6 |
| Baum et al., 2007 (19) | + | + | _ | + | _ | _ | _ | + | + | _ | + | 6 |
| Abbreviations: PEDro: Physiotherapy Evidence Database | | | | | | | | | | | | |
